# Case report: Anti-CNTN1 antibody-associated nodopathies disease with asymmetric onset

**DOI:** 10.3389/fneur.2023.1124540

**Published:** 2023-03-09

**Authors:** Qian Li, Qing Chen, Ting Zhang, Ying Xu, Yanmin Kan, Jing Zhang

**Affiliations:** ^1^Neurology Department, The Third Central Hospital of Tianjin, Tianjin, China; ^2^Tianjin Key Laboratory of Extracorporeal Life Support for Critical Diseases, Tianjin, China; ^3^Artificial Cell Engineering Technology Research Center, Tianjin, China; ^4^Tianjin Institute of Hepatobiliary Disease, Tianjin, China; ^5^The Third Central Clinical College of Tianjin Medical University, Tianjin, China; ^6^The Third Central Clinical College of Nankai University, Tianjin, China

**Keywords:** contactin-1, Ranvier's, autoimmune nodopathies, paranodal, peripheral neuropathy

## Abstract

Anti-contactin-1 (CNTN1) IgG4 antibody-associated nodopathies is an autoimmune antibody-mediated peripheral neuropathy with a unique clinical presentation, pathophysiology, electrophysiology, and therapeutic response. The critical histopathological features are a dense lymphoplasmacytic infiltrate, a storiform pattern of fibrosis, and obliterative phlebitis. Here, a 62-year-old male patient presented with subacute unilateral limb onset, progressive exacerbation, marked weakness of the extremities, cranial, and autonomic nerve involvement. Neurophysiology showed slowed motor nerve conduction velocity (MCV), prolonged distal motor delay (DML), slowed sensory nerve conduction velocity (SCV), decreased sensory nerve activity potential (SNAP) amplitude, decreased amplitude of bilateral neuromotor conduction, abnormal cutaneous sympathetic response (SSR) in both lower extremities, axonal damage, prolonged F-wave latency, and discrete waves. In the initial phase, there was a response to intravenous immunoglobulin (IVIG), and corticosteroids and rituximab were also effective. After 1 year follow-up, the patient improved significantly. This article reports on a patient with nodular disease with anti-contactin-1 (CNTN1) IgG4 antibodies and reviews the literature to improve clinicians' understanding of the disease.

## 1. Introduction

Anti-contactin-1 (CNTN1) IgG4 antibody-associated nodopathies is an autoimmune antibody-mediated peripheral neuropathy. In recent years, some important cell adhesion molecules in the Ranvier's-related region have become the research focus of biomarkers for chronic inflammatory demyelinating polyradiculoneuropathy (CIDP) (1–3). Some scholars put forward the concept of nodo-paranodopathy disease (4, 5) from the perspective of microstructure, however, the latest guidelines (6) clearly state that protein antibody-positive diseases associated with paranodopathy are named autoimmune nodopathies. In this article, we analyzed a patient with positive anti-contactin-1 IgG4 antibody, and reviewed the related literature to improve clinicians' understanding, diagnosis, and treatment of this kind of disease.

## 2. Case presentation

### 2.1. Medical history

A 62-year-old man was admitted for treatment of progressive limb weakness over 4 months. He suddenly felt weakness in the right lower limb during farming over 4 months ago, accompanied by pain and cramps in the calf. He received treatment in the community for 4 days, and the above symptoms disappeared. One week after the initial onset of the weakness, the patient began to have left-sided angle of mouth, salivation, chewing difficulties, difficulty in closing the right eye, slurred speech, transient diplopia, ataxia, weakened tendon reflex of the right limb, and the MRC grade of the right limb muscle was 4 (Table 1), with CT examination showing low-density lesions in the bilateral basal ganglia area. The patient's symptoms progressed after antithrombotic treatment, with recurrence of transient diplopia. He was discharged after 2 weeks of hospitalization. At 9 weeks after the initial onset, after suffering from upper respiratory tract infection, pain occurred in the lower parts of both lower limbs, and the distal knuckles and toes of both lower limbs continued to have numbness. The distal numbness of the limbs gradually spread to the proximal end, accompanied by weakness of both feet, followed by weakness of the limbs, mainly the distal limbs. At 13 weeks after initial onset, he had difficulty in passing stool and needed enema to help, and 15 weeks after initial onset, he had difficulty in passing urine. He was in good health in the past and had a history of COVID-19 vaccination 10 days before the onset of symptoms. He did not have a family history of genetic disease or history of exposure to poisons.

**Table 1:** Medical research council (MRC) scale for muscle examination.

**Functions assessed**
Upper extremity: wrist flexion, forearm flexion, shoulder abduction
Lower extremity: ankle dorsiflexion, knee extension, hip flexion
**Score for each movement**
0—No visible contraction
1—Visible muscle contraction, but no limb movement
2—Active movement, but not against gravity
3—Active movement against gravity
4—Active movement against gravity and resistance
5—Active movement against full resistance
Maximum score: 60 (four limbs, maximum of 15 points per limb)
Minimum score: 0 (quadriplegia)

### 2.2. Admission physical examination

Upon admission, the following were observed: normal cognitive function, the speech was unclear, bilateral forehead lines disappeared, could not wrinkle forehead, bilateral eye closure was weak and right side was obvious, the right nasolabial groove was shallow, the bilateral drum gills were weak, the strength of the right side teeth was weak, the angle of mouth was left, the tongue body was thin, the tongue muscle strength was weak, with right tongue muscle tremor. The masticatory muscle was slightly weak, the pharyngeal reflex disappeared, shoulder shrug and neck were powerful, the neck flexion was weak. Limb muscle strength (MRC) grades: proximal left upper limb: V, distal: II+; left lower limb proximal extensor: IV+, flexor: III, distal: II; proximal right upper limb: V, distal: II+; proximal extensor of right lower limb: IV–, flexor: II+, distal: 0. The muscle tension of both lower limbs decreased, the tendon reflex and abdominal wall reflex of the limbs disappeared. The ataxia of both upper limbs was unstable and both lower limbs cannot be completed. Stocking glove hypoesthesia was observed for vibratory and cold stimuli.

### 2.3. Auxiliary examination

**Figure 1:**
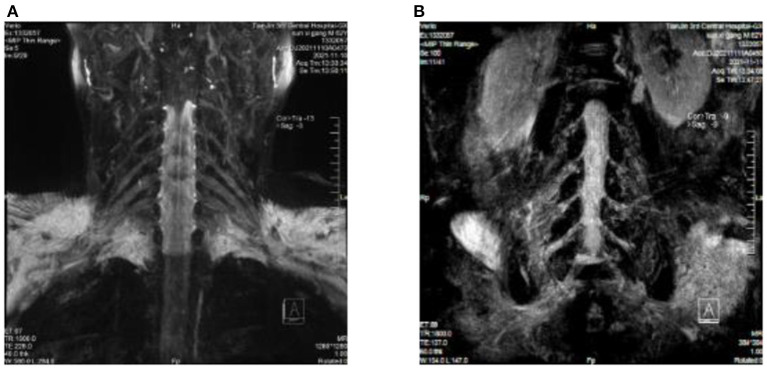
**(A)** Cervical plexus MRI: fluid attenuation inversion recovery (FLAIR) shows slightly thickened bilateral nerve roots at C4/C5–C6/C7. **(B)** Lumbosacral plexus MRI: fluid attenuation inversion recovery (FLAIR) shows bilateral nerve root thickening at the L1/L2–L4/L5 intervertebral space, with slightly higher signal intensity, especially at L2/L3–L4/L5.

**Figure 2:**
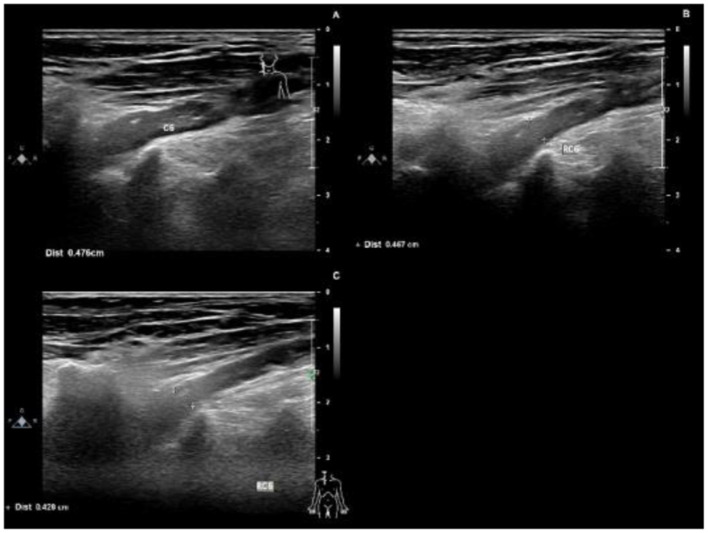
Ultrasounds 4 months after the onset of the disease, the right C6 nerve root was thickened, with a thickness of 0.476 cm **(A)**. 6 months after the onset of the disease, the right C6 nerve root was thickened, with a thickness of 0.467 cm **(B)**. 11 months after the onset of the disease, the right C6 nerve root was thickened, with a thickness of 0.426 cm **(C)**.

Upon auxiliary examination, the following were observed: blood test examination: blood routine, blood coagulation routine; blood glucose, liver and kidney function, thyroid function, tumor markers, serum protein electrophoresis, and vitamin B12 were not significantly abnormal. Cerebrospinal fluid (CSF) protein level: 618.91 mg/dL (normal value 15–45 mg/dL), white blood cell count: 6/μL (normal value 0–8/μL). The neuroelectrophysiology is shown in Table 2. An MRI of the cervical plexus is shown in Figure 1A, an MRI of the lumbosacral plexus in Figure 1B, and musculoskeletal ultrasounds are shown in Figure 2.

**Table 2:** Results of nerve conduction study and F-wave of the anti-contaction-1 IgG4 antibody associated autoimmune nodopathies.

**Nerves**	**Time of onset**			**Normal values**
	**4 months after onset of disease (Left/right)**	**6 months after onset of disease (Left/right)**	**11 months after onset of disease (Left/right)**	
**Median nerve**				
CMAP (mV)	1.2 91%↓/1.4 91%↓	ND/1.0 93%↓	3.5 73%↓/3.1 76%↓	≥7.0
DML (ms)	22.40/21.20	ND/19.43	10.57/10.89	≤ 4.1
MCV (m/s)	18.8 69%↓/18.8 69%↓	ND/20.1 67%↓	40.2 33%↓/36.5 39%↓	≥51.0
SCV (m/s)	ND/32.0 42%↓	34.7 34%↓/ND	26.5 52%↓/30.0 40%↓	≥41.8
SNAP (μV)	ND/5.0 86%↓	2.6 93%↓/ND	1.5 95%↓/2.2 93%↓	≥12.7
F-wave latency (ms)	12.3/ND	ND/27.4	36.4/37.6	≤ 31.0
F-wave occurrence rat (%)	0/0	ND/25	85/75	≥73
**Ulnar nerve**				
CMAP (mV)	5.6 67%↓/4.3 75%↓	ND/2.6 80%↓	5.0 58%↓/5.4 55%↓	≥7.0
DML (ms)	4.58 76%↑/6.09 89%↑	ND/10.78	5.31/5.83	≤ 3.2
MCV (m/s)	–/–	ND/18.1 73%↓	40.0 40%↓/38.0 43%↓	≥56.0
SCV (m/s)	ND/–	–/ND	37.6 31%↓/35.2 35%↓	≥44.2
SNAP (μV)	ND/–	NR/ND	2.3 88%↓/1.5 92%↓	≥6.9
F-wave latency (ms)	71.8/26.9	ND/24.9	ND/36.4	–
F-wave occurrence rat (%)	90/0	ND/45	ND/90	–
**Tibial nerve**				
CMAP (mV)	ND/ 0.3 97%↓	ND/ND	NR/0.3 98%↓	≥4
DML (ms)	ND/ 34.53	ND/ND	ND/6.41 64%↓	≤ 5.1
MCV (m/s)	ND/20.6 64%↓	ND/ND	ND/ND	≥40.5
SCV (ms)	ND/ND	ND/ND	ND/ND	≥35.1
SNAP (μV)	ND/–	ND/ND	NR/NR	≥0.4
F-wave latency (ms)	17.1 /65.4	ND/ND	ND/ND	≤ 51.0
F-wave occurrence rat (%)	10/35	ND/ND	ND/ND	≥80
**Peroneal nerve**				
CMAP (mV)	ND/–	ND/ND	1.2 91%↓/0.7 95%↓	≥3.6
DML (ms)	ND/NR	ND/ND	5.42/6.04	≤ 4.75
MCV (m/s)	ND/–	ND/ND	–/–	≥36.8
SCV (ms)	ND/–	ND/ND	ND/ND	≥46.5
NAP (μV)	ND/–	ND/ND	NR/NR	≥0.8
**Skin sympathetic reflexes**				
DML (ms, Upper limb)	1,350/1,412	NR/NR	NR/NR	–
DML (ms, Lower limbs)	NR/NR	NR/NR	NR/NR	–
**Transient reflex**				
DML (ms)	5.0/5.94	3.65 / 6.51	2.34/2.92	–
CMAP (mV)	0.2↓/0.1↓	0.2↓/ 0.3↓	0.7/0.7	–

ND, not examined; NR, not elicited; CMAP, compound muscle action potential; DML, motor terminal latency; MCV, motor nerve conduction velocity; SCV, sensory nerve conduction velocity; SNAP, sensory nerve action potential; ↑, higher than normal; ↓, lower than normal; –, none.

### 2.4. Admission diagnosis

The diagnosis upon admission was as follows: acute inflammatory demyelinating polyradiculoneuropathy (AIDP)?/ACIDP?/Autoimmune nodopathies? After IVIG treatment, the limb weakness of the patients improved. Blood antibody report: anti-CNTN1 antibody IgG4 positive 1:100, CSF corresponding antibody 1:32. Diagnosis: Autoimmune nodopathies. The treatment was 500 mg methylprednisolone intravenous shock therapy and hormone reduction 5 days after surgery with 48 mg methylprednisolone orally administered. The patient's fatigue symptoms gradually improved, but he was still unable to walk, and improvement of numbness was not obvious.

After discharge, the patient's condition was stable and he was readmitted to the hospital for rituximab treatment because of his inability to walk. Upon follow-up 1 year after onset of disease, he was able to walk normally and felt numbness only in the fingertips. The changes in muscle strength during the three admissions are shown in Table 3. EMG comparisons for the three admissions are shown in Table 2.

**Table 3:** Change in muscle strength between patient admissions.

**(Treatment time/muscle strength)**	**Left upper extremity**		**Left lower extremity**		**Right upper extremity**		**Right lower extremity**	
	**Proximal**	**Distal**	**Proximal**	**Distal**	**Proximal**	**Distal**	**Proximal**	**Distal**
2021.10.21	V	II–III	III	II	V	II–III	II+	0
2021.10.27 (IVIG treatment for 3 days)	V	III	III+	II	V	III	III-	0
2021.10.30 (Hormone therapy for 3 days)	V	III+	IV-	II	V	III+	III	0
2021.11.04	V	III+	IV-	II	V	III+	III	0
2021.11.14	V	III+	IV-	II	V	III+	III	0
2021.12.15	V	IV	IV-	II	V	IV-	III+	I–II
2021.12.22 (Rituximab treatment for 1 day)	V	IV	IV	II	V	IV+	IV	I–II
2021.12.27	V	IV+	IV+	II	V	IV+	IV	II
2022.09.05	V	IV+	IV+	III	V	IV+	IV+	III

## 3. Discussion

The latest guideline (6) names diseases positive for antibodies against Ranviers-related proteins as autoimmune nodopathies and no longer as a CIDP subtype. Positive antibodies to contactin-1 (CNTN1), a cell adhesion molecule at the axial end of the paracord region, may cause nodopathies disease, with unique clinical manifestations, pathophysiological, and electrophysiological changes. Clinical studies found that among patients with nodopathies, the positive proportion of CNTN1 antibody ranges from 2.4 to 6.5% (7–9). In 2018, Hashimoto et al. (10) reviewed 20 patients with CIDP who were positive for anti-CNTN1 antibodies, and reported a relatively high age of onset of 63.0 ± 13.5 years (range 33–81 years, 80% >60 years), with a male-to-female ratio of 2:1. Our patient was an elderly male, consistent with the epidemiological features of the disease. Antecedent infections or vaccinations may also be a trigger for autoimmune nodopathies. In 2022, a NF186+ autoimmune nodopathies case was reported following COVID-19 vaccination (11). Some studies suggest (12, 13) that this may be a result of interactions between the susceptible vaccinated subject and various vaccine components. The mechanisms involved may be molecular patterns, i.e., significant similarities between certain disease-causing elements contained in vaccines and specific human proteins. Our patient developed muscle weakness symptoms after receiving a COVID-19 vaccine, before the onset of the disease, suggesting that COVID-19 vaccination may be the causative agent of CNTN1+, and the speculated mechanism is similar to NF186+, as described above. The clinical manifestation of nodopathies with positive anti-CNTN1 antibody is unique and is different from classical CIDP. CNTN1 antibody-positive patients with nodopathies mostly have subacute onset or rapid progression after chronic onset, mainly manifested as symmetrical proximal and distal limb weakness and sensory abnormalities, which may be accompanied by tremors and sensory ataxia (14–16). The clinical symptoms are closely related to the titer of IgG antibody and the deposition density of IgG in node areas (7). Significant sensory ataxia was found in all patients reported in Japan, but not in patients reported in Spain, suggesting that ethnic or genetic factors may affect antibody-binding epitopes, resulting in clinical heterogeneity (14). Cortese et al. (17) found cranial nerve involvement and respiratory failure in 1 of 3 anti-CNTN1 IgG4 antibody-positive patients. The patient in our case had an unusual subacute unilateral limb onset. The course of the disease was a sudden frustration, manifested as remission-relapse-incomplete remission, and finally progressing to bilateral cranial nerve asymmetry and relative symmetrical limb involvement. Not only was there limb weakness and sensory abnormalities, but also symptoms of cranial nerves and autonomic nerve involvement. The course of the patient was not completely consistent with that reported in other literature. The involvement of cranial nerves and autonomic nerves may be a result of by conduction block, and the mechanism is similar to limb motor nerves.

Protein-cell separation is present in 80–90% of typical CIDP patients. The latest literature suggests that the reference upper limit of cerebrospinal fluid total protein (CSF-TP) should be higher than 45 mg/dl (18). The cerebrospinal fluid (CSF) protein of patients with nodopathies associated with positive anti-CNTN1 antibody is more obvious (16). In addition, MRI of CNTN1 antibody-positive patients may show symmetrical diffuse edema, thickening of nerve roots in the brachial plexus and lumbosacral plexus, as well as edema thickening of nerve roots in musculoskeletal ultrasounds. The mechanism for these may be a lack of blood-nerve barrier at the nerve roots, which are highly susceptible to damage by circulating immune antibodies. The cerebrospinal fluid protein of this patient was 618 mg/dl. MRI and musculoskeletal B-ultrasounds were consistent with the above findings. The musculoskeletal ultrasound showed no significant changes before and after treatment (Table 3). Considering that the improvements observed in auxiliary examinations lagged behind the clinical manifestations, further follow-up observations can be made.

The electrophysiological characteristics of anti-CNTN1 antibody-positive patients with nodopathies were as follows: MCV slowed down, DML prolonged, F wave latency prolonged and conduction block occurred, axonal damage appeared in the early stage, SCV slowed down, and SNAP amplitude decreased (15, 19). The pathological basis of these changes is that the structure of the paranodal region is destroyed, which affects the normal conduction of nerve pulses in myelinated fibers. The electrophysiology of this patient showed that MCV slowed down, DML prolonged, SCV slowed down, SNAP amplitude decreased, bilateral nerves motor conduction amplitude decreased, SSR abnormality for both lower limbs, axonal damage, and F wave latencies prolonged. The neurophysiological manifestations were consistent with those of patients with anti-CNTN1 antibody-positive nodopathies diseases.

The typical pathological manifestations of CIDP were not found in the gastrocnemius nerve biopsy of patients with CNTN1 antibody-positive nodopathies. Destruction of the paranodal structure and separation of the myelin sheath from the axon were observed under an electron microscope (20–22), suggesting that attack by the CNTN1 antibody on the paranodal region is the pathogenesis of the disease.

Most CNTN1 antibodies are mainly IgG4 subtype, with lower binding to Fc receptors, and do not participate in the complement activation pathway (23), with the response to IVIG treatment poor. However, IgG1, IgG2, and IgG3 subtypes can also coexist in CNTN1 IgG4 positive patients, and the latter subtype can induce complementary deposition and activation. Therefore, CNTN1 IgG4 positive patients may respond to IVIG treatment at the initial stage (24). Corticosteroids, plasma exchange, and rituximab are effective in patients with CNTN1 antibody-positive nodopathies disease. According to studies in Japan (14), corticosteroid therapy is effective in 73% of CNTN1 IgG4 positive patients. Plasma exchange improves the treatment rate of CNTN1 antibody-positive patients by removing related antibodies in the plasma. As a targeted drug, rituximab can target the IgG4 antibody, achieving good therapeutic effects by depleting B lymphocytes and downregulating humoral immune responses (25, 26). Our patient had a good response to corticosteroids and rituximab treatment.

## 4. Conclusion

CNTN1 antibody-positive nodopathies is due to the autoimmune antibody attacking the paranodal region of Ranvier, which destroys the composition of the complex in this region and leads to demyelination of myelinated nerve fibers. Patients with CNTN1 antibody-positive nodopathies have unique clinical manifestations, including unilateral limb onset, motor and sensory system involvement, tremor, sensory ataxia, cranial nerve, and autonomic nerve involvement, significantly increased CSF protein, and increased erythrocyte sedimentation rate. MRI and nerve ultrasound showed symmetrical diffuse edema of brachial and lumbosacral plexus nerve roots, while neuroelectrophysiology showed a slowed MCV, prolonged F wave latencies, and conduction block. Axonal damage can occur in the early stage. Most patients respond poorly to IVIG, but some patients respond well to IVIG in the early stage of the disease. Corticosteroids, plasma exchange, and rituximab are all effective in patients with positive CNTN1 antibody. At present, the clinical understanding of paranodal-related antibodies is still insufficient, and standardized treatment can reduce the disability rate and improve the cure rate. Therefore, in the process of diagnosis and treatment of nodopathies diseases, we should screen the antibodies against paranodal proteins in time, to improve the accuracy of diagnosis and guide treatment.

## Data availability statement

The original contributions presented in the study are included in the article/supplementary material, further inquiries can be directed to the corresponding authors.

## Ethics statement

The studies involving human participants were reviewed and approved by the Third Central Hospital of Tianjin. The patients/participants provided their written informed consent to participate in this study. Written informed consent was obtained from the individual(s) for the publication of any potentially identifiable images or data included in this article.

## Author contributions

All authors contributed to the article and agreed to the submitted version.
